# Evaluating the role of amino acids and isothermal dry particle coating in modulating buccal permeation of large molecule drug vancomycin

**DOI:** 10.1038/s41598-024-69144-6

**Published:** 2024-08-24

**Authors:** Anthony Rajabi, Muhammed Idrees, Ayesha Rahman, Affiong Iyire, David Wyatt, Jasdip Koner, Afzal R. Mohammed

**Affiliations:** 1 Aston Pharmacy School, College of Health and Life Sciences, Aston University, Birmingham, B4 7ET UK; 2 School of Healthcare, University of Leicester, Leicester, UK; 3 Aston Particle Technologies Ltd, Birmingham, UK; 4 Dentistry, School of Health Sciences, College of Medicine and Health, University of Birmingham, Birmingham, UK

**Keywords:** Buccal delivery, Biologics, Dry particle coating, Ion pair, Amino acids, Biofilm, Permeation, Vancomycin, Materials science, Techniques and instrumentation, Biological therapy

## Abstract

The formulation and delivery of macromolecules through the oral route pose considerable challenges due to factors such as large molecular weight, pH sensitivity, and limited formulation approaches. This challenge is compounded if the drug is poorly permeable, necessitating innovative drug delivery strategies. Vancomycin, a widely prescribed glycopeptide antibiotic, has an oral bioavailability of less than 10%, leading to predominantly intravenous administration and potential patient discomfort. This study explores the potential of the buccal route as a non-invasive, highly vascularised alternative route of administration, offering a rapid onset of action while bypassing the first-pass metabolism. In this study, vancomycin was coated with L-glutamic acid using an isothermal dry particle coater to modulate permeation through the buccal cell line, TR146. Results confirm significant impact of both amino acid concentration and dry particle coating on the rate and extent of drug permeability. With the introduction of L-glutamic acid and utilisation of the isothermal dry particle coater, vancomycin’s permeation profile increased six-fold compared to the control due to the formation of drug ion-pair complex. Imaging studies showed the presence of layered micronized glutamic acid particles on the surface of dry coated vancomycin particles which confirms the role of dry coating and amino acid concentration in modulating drug permeation. Microbiology experiments in *Staphylococcus aureus*, minimum inhibitory concentration and biofilm disruption studies, provided confirmatory evidence of antimicrobial activity of dry coated glutamic acid-vancomycin ion pair particulate structure. This study demonstrates, for the first-time, buccal delivery of dry coated large molecule drug, vancomycin, through controlled deposition of amino acid using innovative particle coating strategy.

## Introduction

The clinical efficacy and market success of macromolecules have created a significant interest in the development of non-invasive drug delivery strategies for these compounds. This interest is due to the various issues associated with invasive administration such as, patient compliance, storage requirements, and training required for self-administration^1,2^. Among the alternative routes of delivery, the oral route presents a significant opportunity with multiple benefits: ease of administration, established manufacturing methods, availability of targeted drug delivery approaches, wider excipient selection and potentially cheaper cost of production. The buccal route offers several advantages over other conventional routes of administration such as the ability to avoid degradation in the gastrointestinal tract, avoid first-pass metabolism, and high patient acceptability. The high patient acceptability associated with buccal drug delivery is due to its ease of application, pain-free administration, and the convenience it offers for self-medication^3^. Drug absorption in the buccal mucosa mainly occurs via two mechanisms: transcellular and paracellular transport pathways. The transcellular pathway, involves drug molecules passing directly through epithelial cells. The paracellular pathway, involves movement across intercellular spaces between epithelial cells ^4,5^. However, formulating macromolecules for buccal delivery poses significant challenges such as the size and hydrophilic characteristics of macromolecules, stability concerns, electrostatic charge, and potential immunogenicity^6,7^.

The delivery and efficacy of these compounds is also governed by intrinsic solubility and permeability which collectively have a significant impact on the overall bioavailability and efficacy of macromolecule drugs^8^. These parameters are reflected within the biopharmaceutical classification system (BCS), a widely used experimental model that categorises pharmaceutical compounds into four groups (Class I–IV) and can predict in vivo pharmacokinetic performance of these compounds based on measurements of permeability and solubility^8,9^.

**Figure 1:**
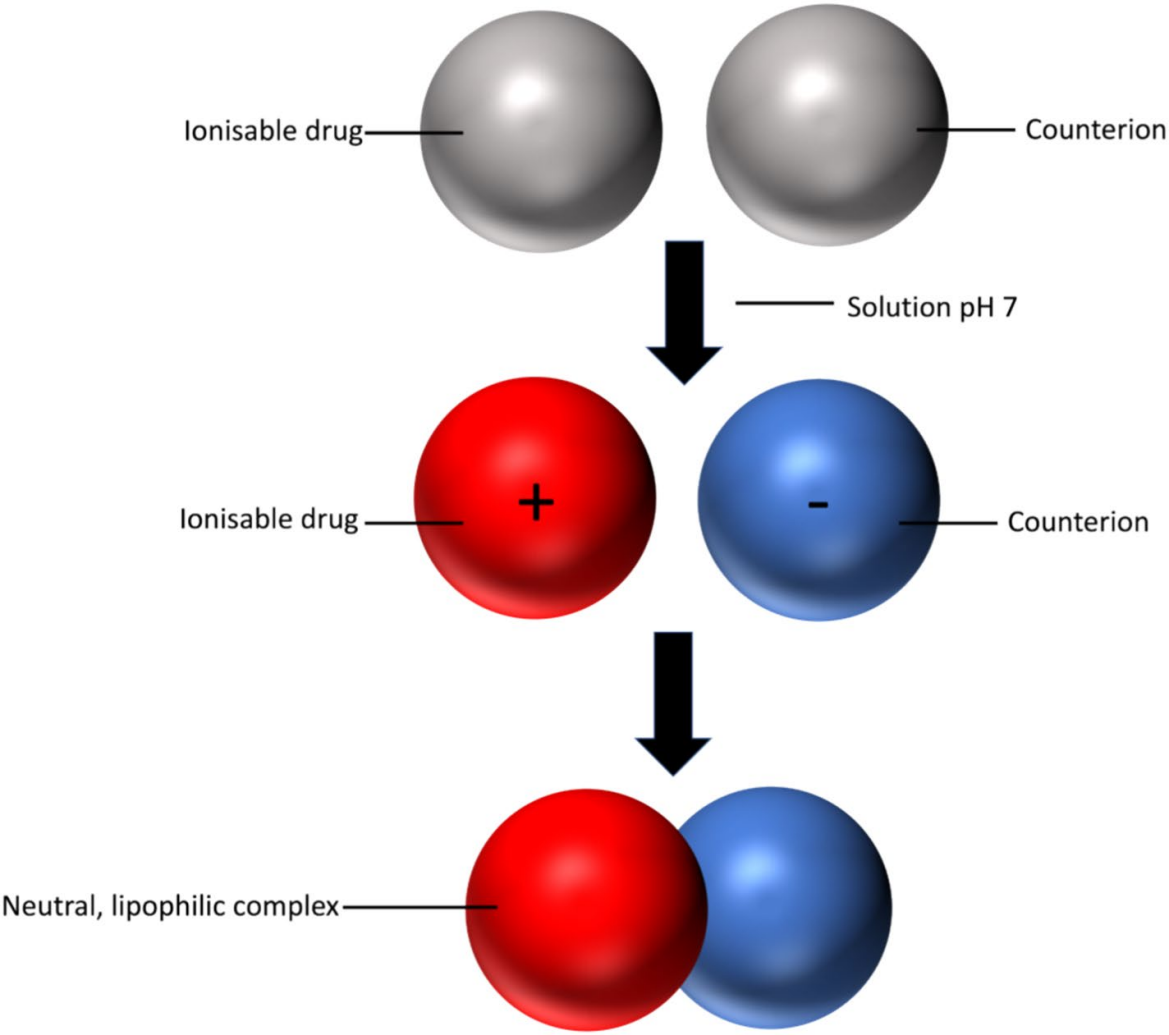
The ion pair hypothesis that an ionisable drug and a counterion can form a more neutral, lipophilic complex depending on the pH of the solution.

BCS class III drugs are categorised as compounds with high solubility, indicated by the highest therapeutic dose which is completely soluble in 250 mL or less of aqueous media, but exhibit low absorption membrane permeability, typically less than 85% of an administered dose^8,10^. BCS class III drugs make up a substantial proportion of the current macromolecules available on the market: particularly peptides and RNA-based medicines^10^. Various strategies have been employed to address the challenge of poor membrane permeability for buccal administration^10^. These strategies include the use of various delivery systems such as: mucoadhesive gels and nanoparticulate systems, microneedles, alongside the utilisation of absorption enhancers or ion-pairing techniques^2,7,11^ Ion pairing is a formulation strategy that consists of combining an ionisable drug and a counter ion that is held together by Coulombic attraction, resulting in the formation of a more lipophilic, neutral complex (Fig. 1). This complex can more readily permeate cell membranes compared to the drug in its original form^6,12^. Upon absorption, the complex dissociates, restoring the drug to its original structure^12^. Careful selection of an appropriate counter ion is crucial, as many are not suitable for pharmaceutical purposes, potentially leading to membrane irritation and toxicity^12^. Evidence supporting the utility of amino acids as counter ions in facilitating insulin transport across the buccal mucosa was provided by our research group, where an increase in the permeation of insulin through the buccal cell line, TR146, was attributed to the inclusion of amino acids. The underlying mechanism involved the formation of potential insulin-amino acid neutral complexes by ion pairing or amino acid mediated transport mechanisms, or a combination of both^6^.

The translation of the founding principles of large molecule buccal transport requires synthesis of a delivery system, preferably as a particulate system to provide controlled and repeatable formulation performance. Vancomycin, a BCS class III glycopeptide macromolecule, was selected as the model compound to evaluate the role of amino acids as ion pairs in the permeation of poorly permeable macromolecules. Vancomycin exhibits poor oral bioavailability, with less than 10% absorbed orally with approximately 80–90% of the drug eliminated primarily through the renal route, necessitating invasive administration^13,14^. Ion pairing as a formulation strategy requires careful consideration of the resultant format in which the ion pair counter ion remains in proximity at the site of absorption and permeation. Here we report for the first time, an isothermal dry particle coater (iDPC™) based on thin layer fluidisation of particles which allows controlled deposition of fine particles over coarse particles, thereby ensuring that the coating material (counter ion) remains in close proximity to elicit the impact of ion pair formation^15^. The iDPC mechanism utilises a high-speed rotating vessel combined with a fluidising nitrogen gas blade, creating a unique thin layer fluidisation mechanism which processes powders in a dry state. In addition, the dry particle coater operates within an isothermal ambient temperature, throughout the entire coating process^16^. This method coats smaller particles onto larger particles without the use of heat, solvents, or physical mixing. In this work, the coating process was investigated by varying process parameters that are associated with the iDPC (flow rate, process time, and centrifugal force). Utilising the iDPC to adequately coat the vancomycin particles with single or multiple layers of amino acids, acting as counter ions, has the potential to enhance ion pair formation within the buccal cavity^17^. The role of the iDPC was evaluated by comparing the permeability of vancomycin across the TR146 buccal cell layers to two controls: a physical mix formulation and vancomycin alone. This study is guided by an overarching hypothesis that isothermal dry coating of fine particles of amino acid, as counterions, on the surface of poorly permeable drug particles produces stable microparticles to modulate buccal permeability without affecting innate functionality of the drug.

## Results and discussion

### Vancomycin-amino acid formulation screening studies

The primary premise of this study was to formulate dry coated particles based on the principle of ion pairing. A systematic approach was implemented in the study design which included a screening phase to identify a suitable amino acid as an adjuvant/counter ion to vancomycin followed by material characterisation, dry coating and permeability studies, and assessment of bacterial activity in microbial cultures.

The objective of the initial permeation study was to determine the impact of two amino acids, L-glutamic acid, and L-histidine, on the quantity of vancomycin that permeated through the TR146 buccal cell line. The selection criteria of amino acids included acid dissociation constant (pKa) values, that had a potential to form an ion pair complex with vancomycin or activate carrier mediated processes^18,19^. L-glutamic acid and L-histidine were chosen based on their specific pKa values (L-glutamic acid pKa values = 2.19, 9.67, 4.25; L-histidine pKa values = 1.92, 9.17, 6.04)^20,21^. These pKa values are pivotal as they dictate the ionisation state of the amino acids, thereby influencing the ability to form ion pairs with vancomycin, depending on the pH of the solution.

Following the selection of the amino acids, two formulations were prepared by physically mixing the vancomycin and amino acid. A TR146 buccal permeation study was then conducted to compare the formulations against the control, which consisted of just vancomycin. One formulation contained vancomycin and L-glutamic acid, while the other consisted of vancomycin and L-histidine, with both formulations having a concentration of 90% vancomycin and 10% amino acid. These results of permeation study offer valuable insights into the impact that both amino acids have in augmenting the permeation of vancomycin across the buccal mucosa.

The formulation containing vancomycin and L-glutamic acid demonstrated a significant increase in vancomycin that permeated the TR146 buccal cell layers compared to the control (ANOVA, *P* = 0.0022, data not shown). This increased permeation could potentially be attributed to ion pair formation. In a solution at pH 7, vancomycin molecules carry a positive charge, while L-glutamic acid molecules carry a negative charge. This is attributed to the ionisation of vancomycin's three ionisable sites (pKa values = 9.6, 10.4, 12), rendering the acidic site (pKa = 2.18) over 99% ionised, and the two basic sites (pKa = 7.8, 8.9) 50–90% and 90–99% ionised, respectively^22^. In a solution at pH 7, L-glutamic acid can be assumed to possess a negative charge, due to its ionisable sites being over 99% ionised^21^. As a result, vancomycin and L-glutamic acid possess an overall positive and negative charge, respectively, promoting ion pairing^23^. The counter ions may form a more lipophilic, neutral ion pair complex through Coulombic attraction, facilitating more efficient permeation through the buccal mucosa. The permeation pathway could either be through transcellular passive diffusion or possibly via amino acid nutrient transporters on cell membranes^24,25^.

The vancomycin and L-histidine formulation did not exhibit a significant difference in permeation of vancomycin when compared to the control (*P* = 0.0548). In a solution with pH 7.46, this formulation permeated less vancomycin compared to both the L-glutamic acid formulation and the control. The ionisation of vancomycin was similar across both formulations. The reduced amount of vancomycin permeation may be attributed to L-histidine's α-carboxyl group (pKa = 1.82) and α-ammonium group (pKa = 9.17) being 90–99% ionised, while the weak basic side chain (pKa = 6.04) showing an ionisation between 1 and 10%. As a result, L-histidine molecules are predominantly neutral, prohibiting the formation of an ion pair with vancomycin, which may explain the decreased permeation compared to the other formulations^12,18^.

Based on the results, L-glutamic acid was chosen for further investigation to evaluate the influence of particle size and the impact of dry particle coating on the permeability of vancomycin. Trans-epithelial electrical resistance (TEER) was measured before and after the permeation studies and there was no significant change (*P* > 0.05).

### Characterisation of vancomycin and L-glutamic acid

Pre-formulation characterisation requires a thorough understanding of the starting material to enable formulation optimisation. A systematic approach to material characterisation involves evaluating properties such as particle size, thermal profile, and morphology. Prior to evaluating the feasibility of dry coating, it is vital to understand particle size distribution of the material. Dry particle coating exploits differences in particle size distribution and controlled coating is achieved when there is at least a two-fold difference in particle size between the carrier (material to be coated) and the coating material (material that is layered on the carrier).

**Figure 2:**
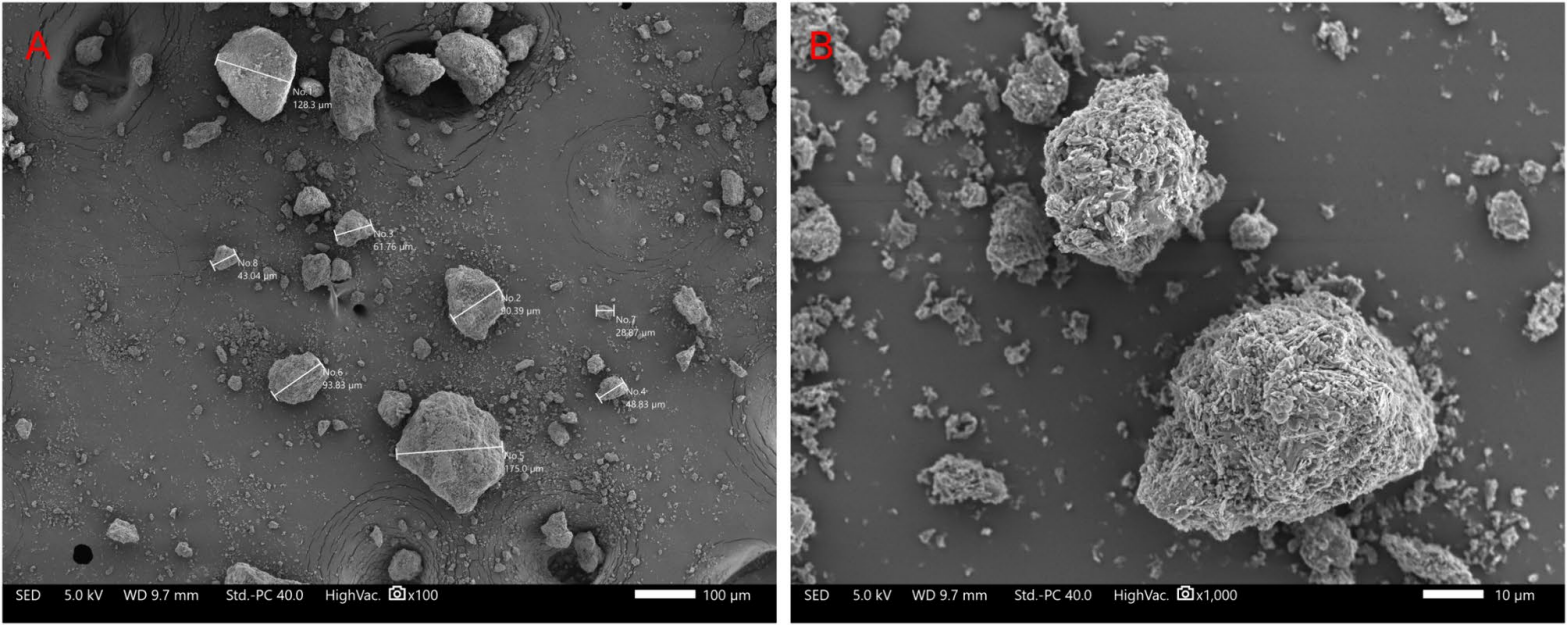
(**A**) SEM illustrating the size distribution of the vancomycin particles, with the largest particle in this image being 175µm and the smallest being 20µm. (**B**) SEM image illustrates the morphology associated with vancomycin particle at a higher magnifications.

Particle size analysis of vancomycin, and L-glutamic acid was conducted using laser diffraction, with the volume mean diameter (VMD) being 51.64 µm and 62.61 µm, as presented in Supplementary Table S1. In addition, SEM images were taken to confirm the particle size analysis and to determine particle morphology. Figure 2A displays the particle size distribution of the vancomycin particles, which aligns with the results obtained from laser diffraction particle size analysis. Vancomycin consists of a large particle distribution profile which includes a significant proportion of fine particles as evidenced in SEM and confirmed by laser diffraction. The SEM image in Fig. 2B, illustrates the morphology of vancomycin revealing particles with a tile-like shape that appear to interlock, forming agglomerates.

The particle size distribution revealed that L-glutamic acid had a volume mean diameter (VMD) slightly larger than vancomycin. This initial characterization of particle size distribution is crucial before coating, as the goal is to effectively coat vancomycin particles with amino acids to facilitate ion pair formation and minimise the risk of dissociation upon dilution.

**Figure 3:**
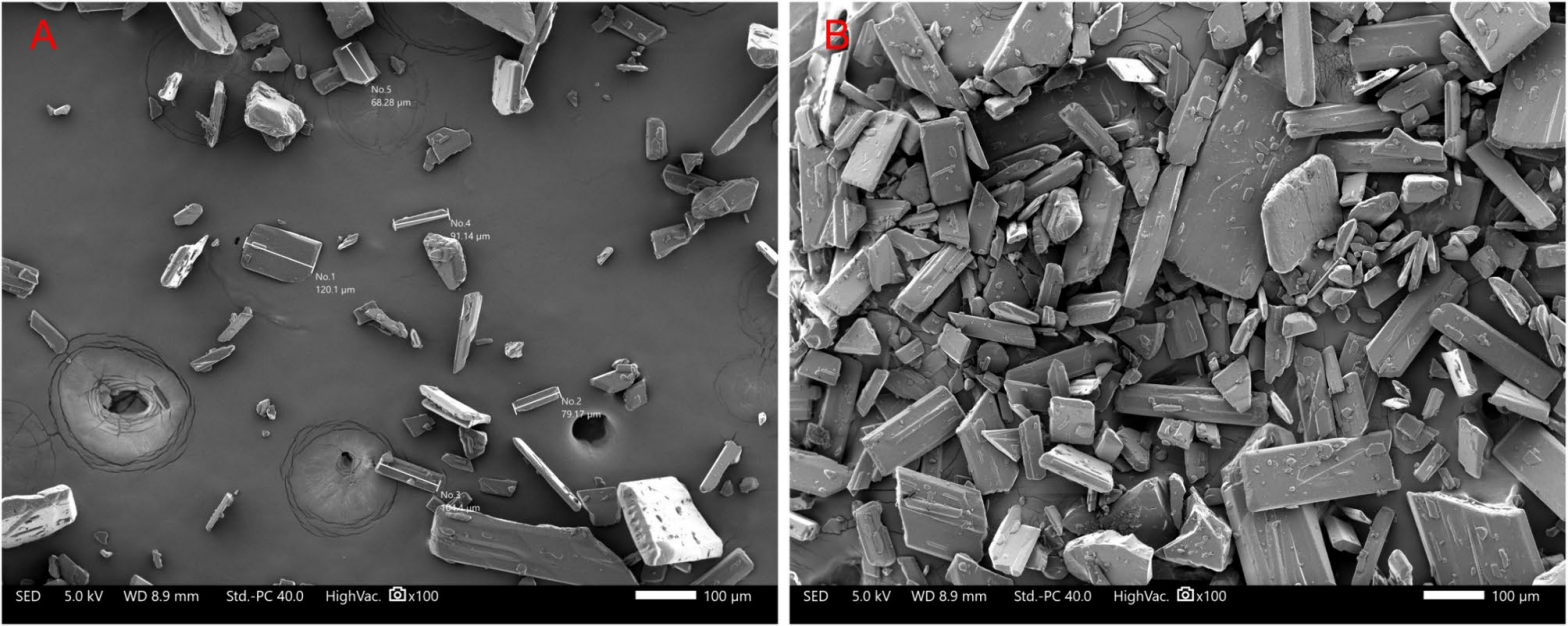
(**A**) SEM image illustrating the particle size distribution of L-glutamic acid, and (**B**) SEM image illustrates the morphology of L-glutamic acid.

Further SEM imaging was conducted to validate the particle size distribution of L-glutamic acid as determined by laser diffraction and can be seen in Fig. 3A. Additionally, Fig. 3B shows the L-glutamic acid particle morphology revealing a rectangular, brick-like structure.

### L-glutamic acid particle size reduction by ball milling

As mentioned above, the aim was to coat the vancomycin particles with L-glutamic acid, allowing for a more controlled L-glutamic acid particle deposition to facilitate ion pair formation, thereby enhancing vancomycin permeability. Particle size analysis completed by laser diffraction shows that the particle size distributions of L-glutamic acid and vancomycin are similar, requiring a reduction in particle size of L-glutamic acid to meet the requirement for the ‘guest’ particles to be 2–3 times smaller than the ‘host’ particles. This requirement is a key factor in successful coating processes with the iDPC^16^.

In order to decrease the particle size of L-glutamic acid, a planetary ball mill was employed. A planetary ball mill comprises a hollow cylinder that can be rotated on its horizontal longitudinal axis, containing agate balls occupying 30–50% of its total volume^26^.

**Figure 4:**
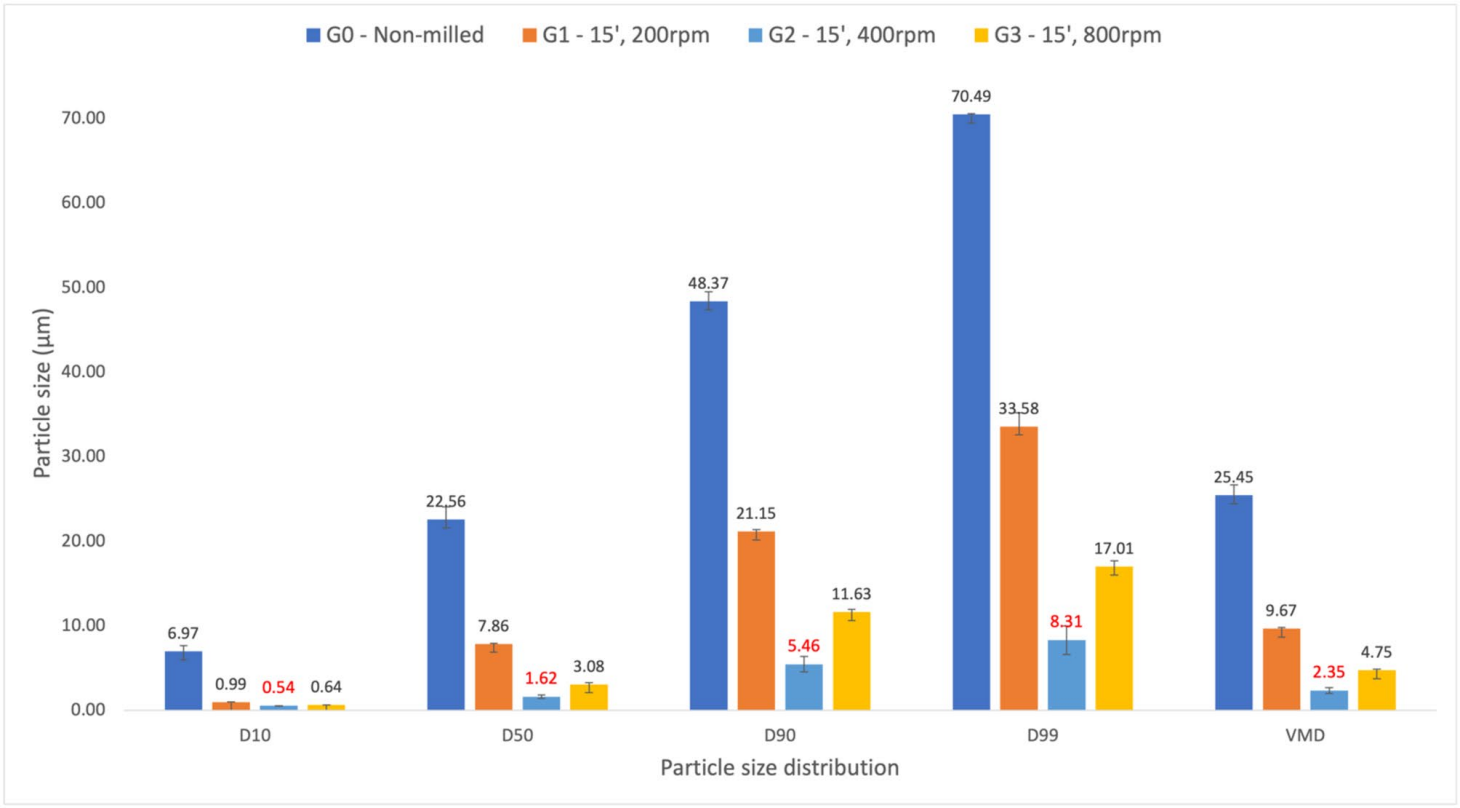
A comparison of the particle size analysis (n = 3) of L-glutamic acid that had been processed at three different speeds using a ball to powder ratio (BPR) of 8. G0 was non-milled, which served at the control. G1 milling parameters: 15-min run time, 200rpm, G2 milling parameters: 15-min run time, 400rpm, G3 milling parameters: 15-min run time, 800rpm.

**Figure 5:**
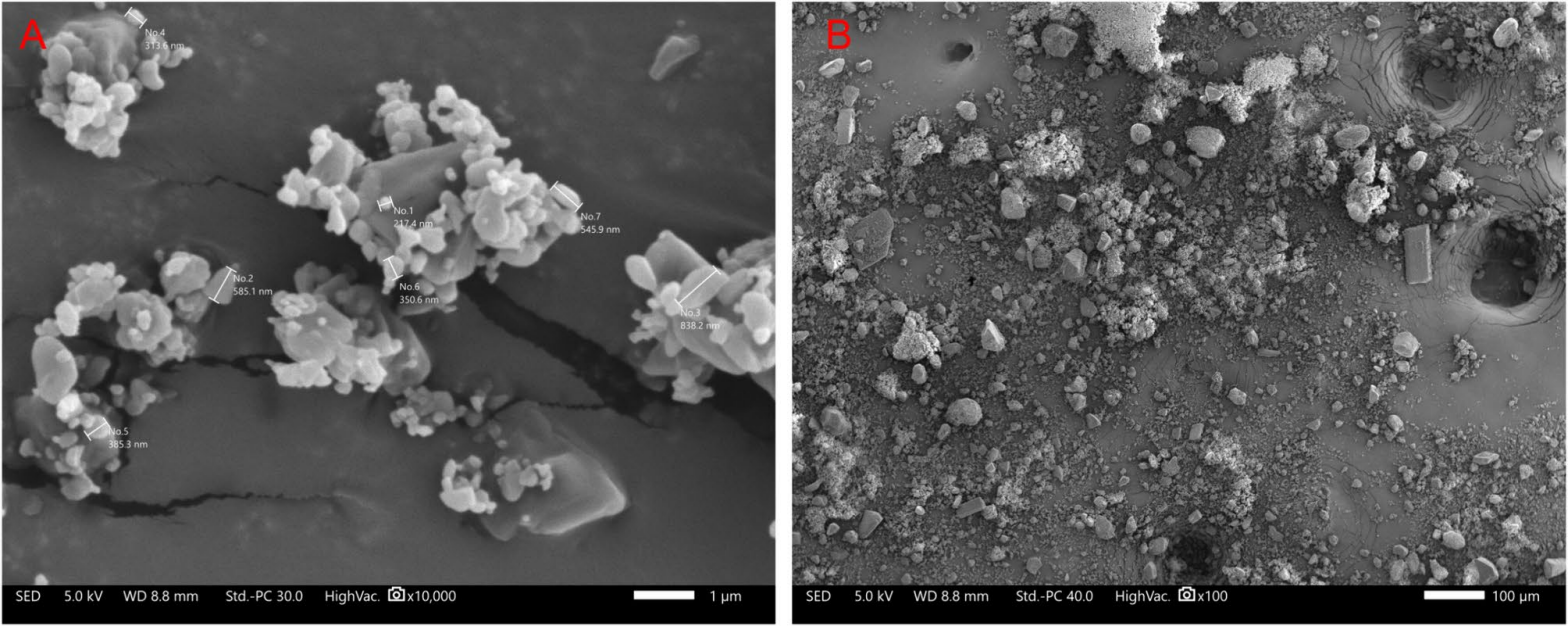
(**A**) SEM image illustrating the particle size distribution of ball milled L-glutamic acid, and (**B**) SEM image illustrates the morphology of the ball-milled L-glutamic acid. The process parameters associated with the ball milled L-glutamic acid: 15 min run time, 400 rpm, and 8 ball to powder ratio.

To optimise the particle size reduction, various ball milling parameters were evaluated to achieve an L-glutamic acid particle size 2–3 times smaller than that of vancomycin, aiming for a VMD of approximately 3µm. The specific parameters, along with the corresponding particle size analysis for the different milled powders, are presented in Fig. 4. To further evaluate the impact of ball milling on L-glutamic acid, SEM images were captured to compare and determine any changes in particle morphology of the L-glutamic acid particles as seen in Fig. 5. Figure 5A shows a clear reduction in L-glutamic acid particle size distribution compared to the non-milled L-glutamic acid. Additionally, Fig. 5B shows a change in particle morphology with the ball milled L-glutamic acid exhibiting a more powder-like appearance with a circular shape when compared to quadrilateral shape as seen in the non-milled form (Fig. 3B).

Upon reviewing the particle size analysis post ball milling, batch G2, as depicted in Fig. 4, was selected, as the particle size distribution aligns well with the recommended ratio when using the iDPC to coat one powder onto another, in a single or multiple layers. The milling parameters associated with batch G2 included a run time of 15 min, a speed set at 400 rpm, and a ball to powder ratio of 8. However, a significant challenge with particle size reduction is the propensity of the fine particles to agglomerate over time^27^. This tendency can be attributed to surface energetics which arise as a consequence of substantive increase in surface area and the creation of high-energy sites, resulting in an overall increase in surface energy^28^.

To ensure the size stability of the ball milled L-glutamic acid particles, a time-trial stability study was conducted over a period of 28 days. The findings indicated no significant change in size (ANOVA, *P* > 0.05), as presented in Supplementary Table S2. This assessment aimed to confirm that the particle size of the ball-milled L-glutamic acid remained consistent over time—a key factor in maintaining consistent formulation performance.

### Dry coated formulations and Permeation study

The iDPC technology offers several adjustable process parameters that may influence the characteristics of the resulting formulation. These parameters include flow rate (measured in litres per minute), rotational speed (in revolutions per minute), and the duration of the process (in minutes). The functionality of the iDPC incorporates three simultaneously occurring phases when the powders are introduced into the system: de-agglomeration, dispersion, and adsorption. Each of the phases can play a role in determining the extent and uniformity of the carrier particles coating the host particles.

Three different concentrations of L-glutamic acid and vancomycin (10:90, 45:55, and 80:20 L-glutamic acid: vancomycin) were examined to determine the impact of varying L-glutamic acid amounts on vancomycin's release profile. This wide range of concentrations aimed to investigate the feasibility of coating vancomycin particles with varying particle densities of L-glutamic acid. Specifically, for formulations with 80:20 and 45:55 (L-glutamic acid: vancomycin) ratios, the aim was to investigate the potential coating of multiple layers of L-glutamic acid on vancomycin particles and its impact on permeation. Conversely, the 10:90 (L-glutamic acid: vancomycin) formulation was chosen to assess whether similar permeation profiles could be achieved even with a lower concentration of L-glutamic acid.

**Figure 6:**
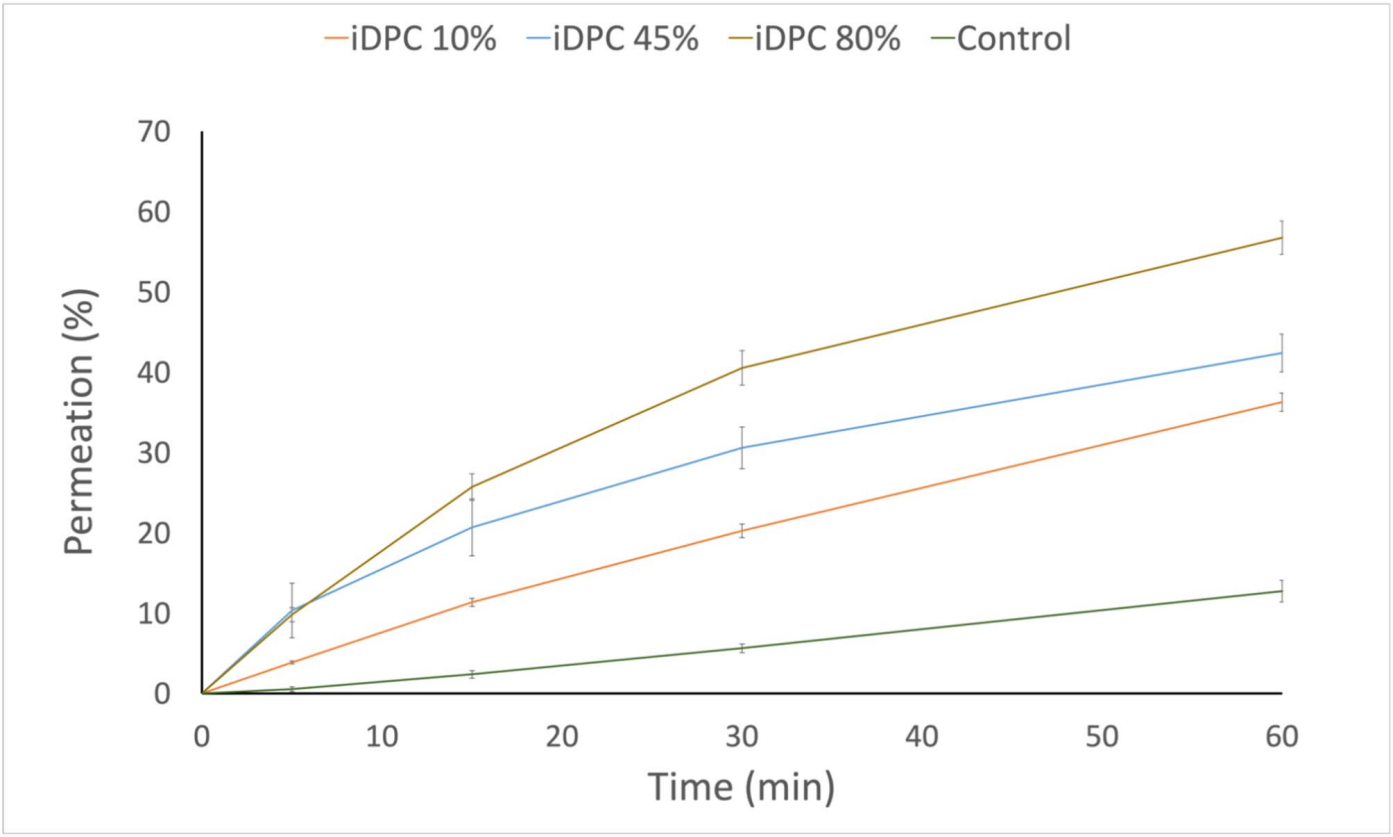
TR146 permeation study results of formulations and the control (vancomycin) over 60 min (n = 3). The process parameters of formulations iDPC 10%, iDPC 45%, and iDPC 80% are as follows: 2.5 min of pre-processing time, 17.5 min of process time, 22.5l/min flow rate, 95 relative centrifugal force (RCF).

These three formulations were developed utilising the iDPC, with identical process parameters maintained across all formulations (17.5 min process time, 22.5 l/min, 95 centrifugal force) with varying concentrations of L-glutamic acid were incorporated. While to ensure consistency in the permeation study, an equivalent quantity of vancomycin was calculated for each formulation. The TR146 permeation study was completed in triplicates (n = 3), the results can be seen in Fig. 6.

The results from the permeation study, as depicted in Fig. 6, illustrate a significant impact of L-glutamic acid and its concentration on the permeation of vancomycin through TR146 buccal cell layers. Notably, the introduction of L-glutamic acid at concentrations of 10%, 45%, and 80% resulted in a significant impact on the quantity of vancomycin permeating the TR146 cell layers when compared to the control group (*P* < 0.0001).

Furthermore, statistical analysis revealed significant variations in the vancomycin permeation profiles among the different formulations with varying concentrations. This observation highlights the significant role played by the concentration of L-glutamic acid in modulating the permeation profile of vancomycin.

The variation in permeation profiles among the formulations offers an interesting insight into the role of L-glutamic acid concentration. iDPC 80% demonstrated the highest permeation of vancomycin, followed by iDPC 45% and then iDPC 10%.

The variation in permeation profiles observed between iDPC 80%, iDPC 45%, and iDPC 10%, seen in Fig. 6, suggests the presence of a more comprehensive coverage of L-glutamic acid on the vancomycin particles as the concentration increases. This more complete coverage is likely to prevent ion pair dissociation, thereby improving the permeability. SEM images of the formulations, iDPC 10%, iDPC 45%, and iDPC 80%, can be seen in Fig. 8A–C, respectively. The images distinctly illustrate the increase in L-glutamic acid coverage on the vancomycin particles within the formulations as the concentration of L-glutamic acid increases.

In addition to the impact of L-glutamic acid concentration, the size reduction of the ball milled L-glutamic acid could also play a significant role. The particle size of the ball-milled L-glutamic acid had been reduced to 4% of its original size pre-ball mill. This reduction in particle size can enhance the adsorption of L-glutamic acid onto the vancomycin particles^29^. Reducing the particle size of L-glutamic acid increases its surface area, allowing the L-glutamic acid to more effectively and uniformly coat the vancomycin particles by conforming to the vancomycin particle shape^30^. This facilitates a more comprehensive coverage of the vancomycin particles, potentially increasing the likelihood of ion pair formation and reducing the probability of ion pair dissociation.

**Figure 7:**
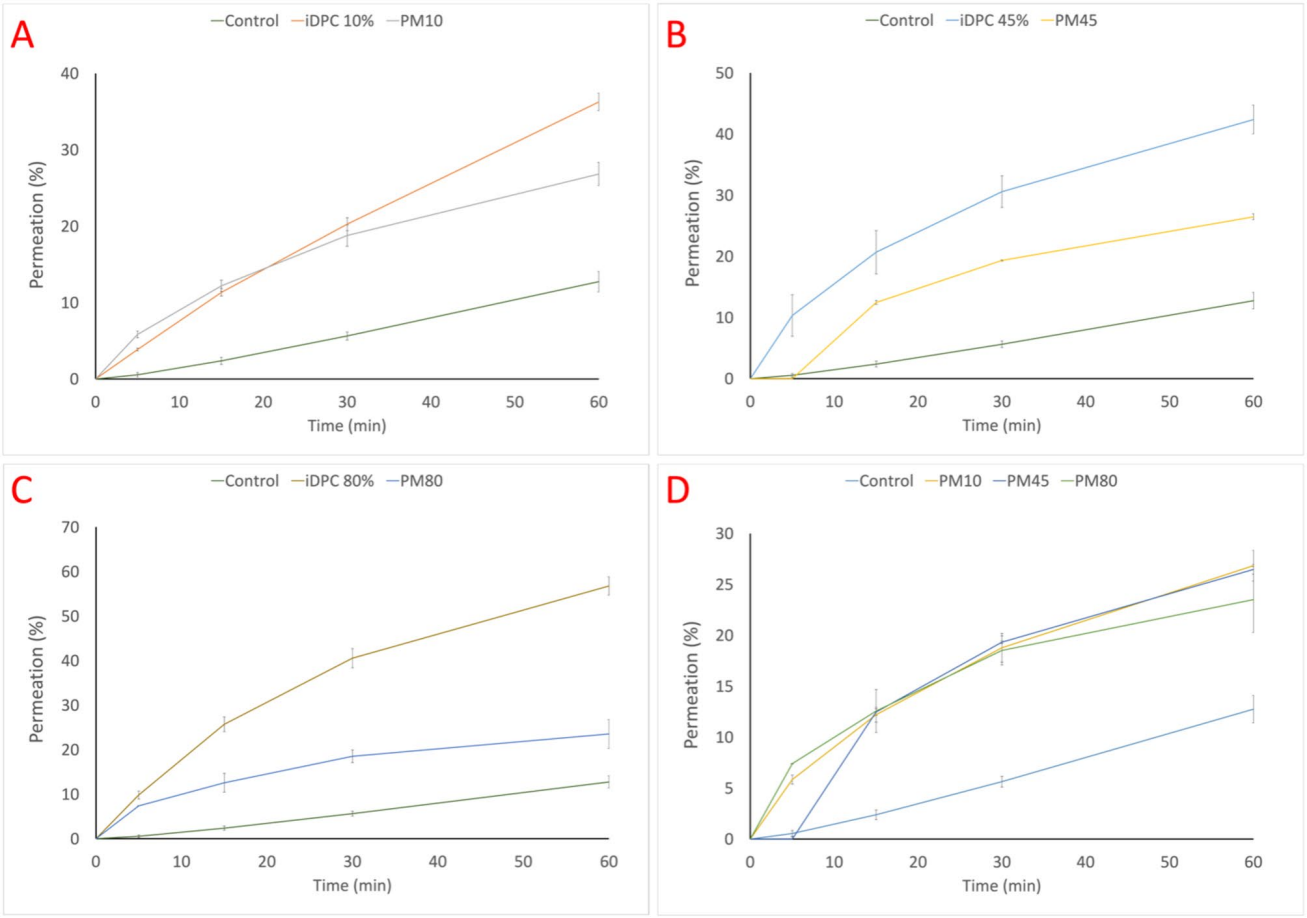
TR146 permeation study results of formulations and their physical mix counter parts: (**A**) shows the permeation profiles of the iDPC vancomycin formulation containing 10% L-glutamic acid in comparison to the physically mixed vancomycin formulation with 10% L-glutamic acid (n = 3). (**B**) Permeation profiles of the iDPC vancomycin formulation containing 45% L-glutamic acid in comparison to the physically mixed vancomycin formulation with 45% L-glutamic acid (n = 3). (**C**) Permeation profiles of the iDPC vancomycin formulation containing 80% L-glutamic acid in comparison to the physically mixed vancomycin formulation with 80% L-glutamic acid (n = 3). The process parameters of the iDPC formulations are as follows: 2.5 min of pre-processing time, 17.5 min of process time, 22.5 l/min flow rate, 95 relative centrifugal force (RCF). (**D**) Permeation profiles of physically mixed formulations of 10%, 45%, and 80% L-glutamic acid (n = 3).

To determine the specific impact of the iDPC on enhancing permeation, as opposed to the influence of L-glutamic acid concentration on vancomycin permeation, an additional permeation study was conducted. This study aimed to compare the permeation profiles of formulations achieved through physical mixing with those prepared using the iDPC. The physically mixed formulations were prepared to match the vancomycin and L-glutamic acid concentrations present in their iDPC counterparts. The physical mixing process involved a 5-min blending of vancomycin and L-glutamic acid using a spatula. The results of this comparative analysis are presented in Fig. 7.

As evident from Fig. 7, notable distinctions in permeation profiles are apparent across the tested concentrations. In Fig. 7A, the 60-min permeation profiles of iDPC 10% L-glutamic acid, physically mixed formulation (10% L-glutamic acid), and the control (vancomycin alone) are depicted.

The statistical comparison between iDPC 10% formulation and the physical mix (10% L-glutamic acid) revealed a significant difference (*P* = 0.0005). Similarly, in Fig. 7B,C, the permeation profiles over 60 min for iDPC 45% L-glutamic acid and iDPC 80% L-glutamic acid, respectively, are compared with their physically mixed counterparts and controls. Statistical analyses demonstrated significant disparities, with P-values of < 0.0001 and < 0.0004, respectively.

**Figure 8:**
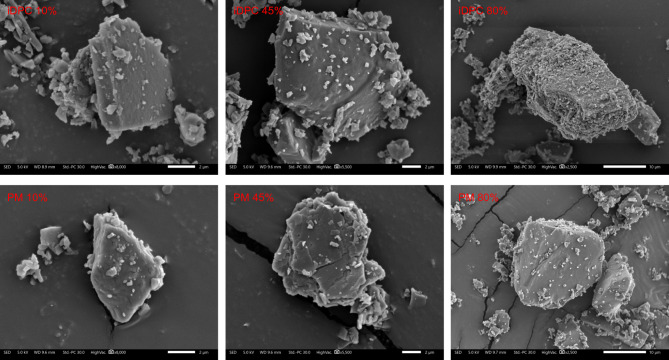
SEM images of iDPC 10%, 45%, and 80% formulations on the top row (left to right) and SEM images of physically mixed formulations 10%, 45%, and 80% L-glutamic acid. The process parameters of the iDPC formulations are as follows: 2.5 min of pre-processing time, 17.5 min of process time, 22.5 l/min flow rate, 95 relative centrifugal force (RCF).

SEM images of the physical mix formulations compared to the iDPC formulations can be seen in Fig. 8. The images clearly show the difference between the physically mixed formulations in comparison to their iDPC formulated counter parts regarding the coverage of L-glutamic acid on the vancomycin particles. However there appears to be little or no difference in L-glutamic acid coating within the physically mixed formulations. This observation aligns with the findings of the statistical analysis conducted on the permeation study which confirmed no significant difference in permeation profiles among the physically mixed formulations.

This study establishes that the iDPC-coated vancomycin, across various L-glutamic acid concentrations, markedly influences the quantity of vancomycin permeating through TR146 buccal cell layers in comparison to their physical mixed counterparts and the control.

The difference in release profiles between the iDPC formulations and the physical mix formulations observed in Fig. 7 can potentially be attributed to the varying degrees of centrifugal force and potential fluidisation within the iDPC drum, as modulated by the drum rotational speed and nitrogen gas flow rate. The centrifugal force increases with the rotational speed of the drum, exerting greater force on the particles and thereby promoting deagglomeration^31,32^. The enhanced force can also increase the likelihood of particle-to-particle collisions, facilitating a more effective coating process potentially due to better adhesion between the particles, which can be seen in Fig. 8^33^. Miyazaki et al. (2019) demonstrated that agglomerates of atropine sulphate were effectively broken down and dispersed onto lactose particles when processed in a centrifugal mixer^34^. Fluidisation, achieved by introducing a flowing gas, allows particles to suspend in a fluid-like state, facilitating homogenous mixing^35^. The degree of fluidisation is thought to be directly influenced by the rate of gas flow. If the flow rate is too low or non-existent, fluidisation will not occur, leading to diminished mixing of the particles, while an excessively high flow rate can result in excessive entrainment, resulting in loss of material^36^.

As discussed earlier, the L-glutamic acid concentration had an impact on vancomycin permeation when looking at formulations prepared in the iDPC. However, Fig. 7D shows no significant difference in permeation profiles among the physically mixed formulations containing 10%, 45%, and 80% L-glutamic acid. While there was a clear distinction in permeation profiles between formulations with differing L-glutamic acid concentrations in the iDPC-prepared formulations, this difference was not observed in physically mixed formulations. This further highlights the impact of the iDPC and the role of centrifugal force and fluidisation in sufficiently coating the vancomycin particles with L-glutamic acid. Despite this, there was still a significant difference in the permeation profile when compared to the control, indicating some impact and potential ion pair formation in the physically mixed formulations (*P* = 0.0032). TEER was measured before and after the permeation studies and there was no significant change (*P* > 0.05).

**Figure 9:**
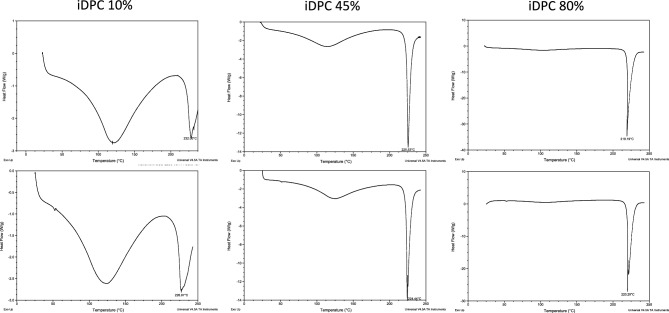
DSC thermograms for day 0 on the top of the figure and DSC thermograms for month 4 underneath the respective formulations; each sample was ramp heated at 25 °C/minute from 25 to 250 °C.

Additionally, to investigate whether these formulations would remain stable, a 4-month stability test was undertaken, where formulations were kept in a 25 °C cabinet set to 60% humidity, as suggested by the ICH guidelines^37^. Three formulations containing three different concentrations of L-glutamic acid (10%, 45%, and 80%) were chosen for stability testing. The tests undertaken to determine stability were differential scanning calorimetry (DSC) and content uniformity. The results of the DSC and content uniformity testing indicated no significant changes from day 0 to end of month four. The relative standard deviation (RSD) for content uniformity did not surpass 2% (Table 1) and DSC thermograms comparing day 0 to month 4 can be seen in Fig. 9.

**Table 1:** Relative standard deviation calculated from content uniformity data over 4 months completed by HPLC analysis for three iDPC formulations containing 10%, 45%, and 80% L-glutamic acid.

Formulation	Month 0 (%RSD) (%)	Month 1 (%RSD) (%)	Month 2 (%RSD) (%)	Month 4 (%RSD) (%)
iDPC 10%	0	1	0	0
iDPC 45%	1	2	1	2
iDPC 80%	4	1	2	2

The process parameters of the iDPC formulations are as follows: 2.5 min of pre-processing time, 17.5 min of process time, 22.5 l/min flow rate, 95 relative centrifugal force (RCF).

### Antimicrobial activity and biofilm assay

Following the evaluation of dry coating on permeability, it was important to ascertain whether particle coating impacted the antibacterial activity, as a measure of activity/efficacy of vancomycin. To assess the impact of iDPC, and the inclusion of L-glutamic acid on the antibacterial activity of vancomycin, several experiments were conducted using *S. aureus* as the target microorganism. Vancomycin has been used routinely to effectively treat *S. aureus* infections for over five decades^38^. The experiments conducted included minimum inhibitory concentration (MIC), minimum lethal concentration (MLC) test and antibiofilm activity (dispersal and inhibitory activity).

The MIC test is a common antimicrobial assay and was conducted to ascertain the lowest concentration of vancomycin that would completely inhibit visible growth or turbidity of *S. aureus*^39^. Subsequently, a MLC test followed, which is defined as the lowest concentration that kills at least 99.9% of bacterial cells^40^. In this study, MIC and MLC were performed for three iDPC formulations featuring varying L-glutamic acid concentrations (10%, 45%, and 80%), conducted on the *S. aureus*. All formulations were processed by the iDPC, maintaining consistent process parameters. Additionally, MIC and MLC evaluations were conducted for neat vancomycin and iDPC-processed vancomycin, this was to ascertain whether the iDPC had impacted the antibacterial activity of vancomycin.

The results of the MIC and MLC, presented in Table 2, demonstrated consistent values across all the formulations, including neat vancomycin and iDPC-processed vancomycin.

**Table 2:** MIC and MLC results illustrating the minimum inhibitory concentration for the control (vancomycin unprocessed by iDPC), processed vancomycin, iDPC 10%—a 10:90 L-glutamic acid: vancomycin formulation, iDPC 45%—a 45:55 L-glutamic acid: vancomycin formulation, iDPC 80%—a 45:55 L-glutamic acid: vancomycin formulation. The process parameters of the iDPC formulations are as follows: 2.5 min of pre-processing time, 17.5 min of process time, 22.5 l/min flow rate, 95 relative centrifugal force (RCF).

Formulation	MIC (μg/mL)	MLC (μg/mL)
Control	3.125	6.25
Processed vancomycin	3.125	6.25
iDPC 10%	3.125	6.25
iDPC 45%	3.125	6.25
iDPC 80%	3.125	6.25

The MIC and MLC values of the formulations were within the acceptable range for vancomycin treatment according to the Clinical and Laboratory Standard Institute (CLSI), indicating that the inclusion of L-glutamic acid and the iDPC process did not affect the antibacterial activity of vancomycin^41^. The MIC and MLC values are consistent with those reported in literature^42^.

Biofilms are microbial communities that adhere to a wide variety of surfaces, enclosed in a self-produced matrix of extracellular polymeric substances (EPS), consisting of polysaccharides, proteins, and DNA^43,44^.

Biofilm formation progresses through five stages: reversible attachment of planktonic bacteria, followed by irreversible attachment facilitated by cell adhesion structures like pili and fimbriae. Subsequent EPS productions leads to the formation of microcolonies, creating a complex, three dimensional biofilm matrix ^44–46^. The final stages involve biofilm maturation through cell division and recruitment of additional bacteria, culminating in dispersion of bacteria from the biofilm to initiate new biofilms^45,46^. The coordination of biofilm formation occurs through cell-to-cell communication process, called quorum sensing^47^. Quorum sensing enables bacteria to regulate gene expression and biofilm development by detecting the accumulation of specific signalling molecules^48^.

Bacterial biofilms pose a formidable challenge when combatting antibacterial resistance. Once attached, bacteria within the biofilm exhibit a substantial resistance rendering them 10–1000 times less susceptible to antimicrobial agents compared to their planktonic counterparts^49,50^. Consequently, significant attention is directed towards developing strategies to treat and prevent the formation of biofilms. The strategies can be categorised into the following approaches: inhibition of biofilm formation, weakening of the existing biofilms, disruption or dispersal of biofilm structures, and targeting the bacteria within biofilm subpopulations^44^.

**Figure 10:**
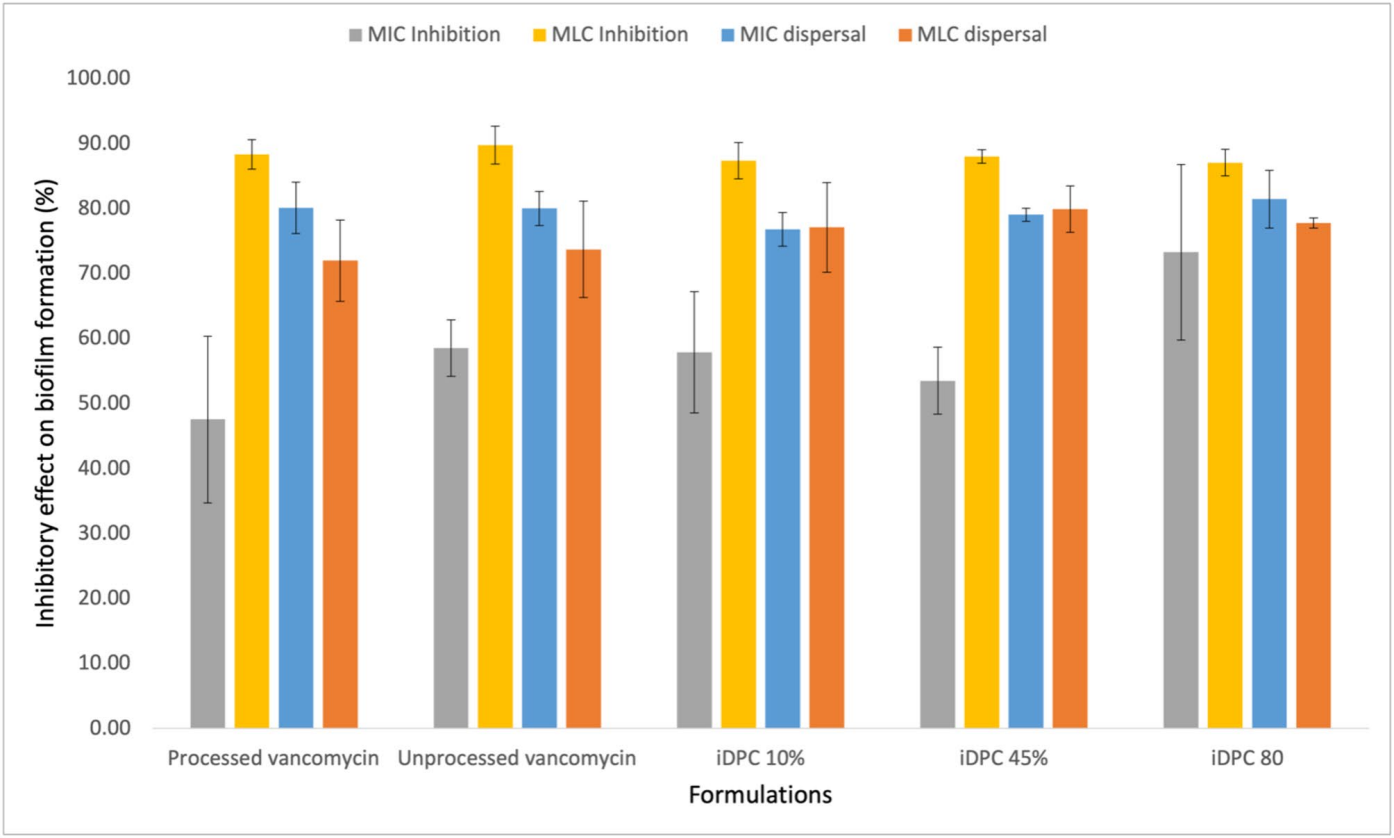
Dispersal and inhibition of *S. aureus* biofilm using processed vancomycin (20 min, 95 RCF, 22.5 l/min), unprocessed vancomycin, iDPC 10%, iDPC 45%, and iDPC 80%. The process parameters of the iDPC formulations are as follows: 2.5 min of pre-processing time, 17.5 min of process time, 22.5 l/min flow rate, 95 relative centrifugal force (RCF). D.) shows the permeation profiles of physically mixed formulations of 10%, 45%, and 80% L-glutamic acid (n = 3).

**Figure 11:**
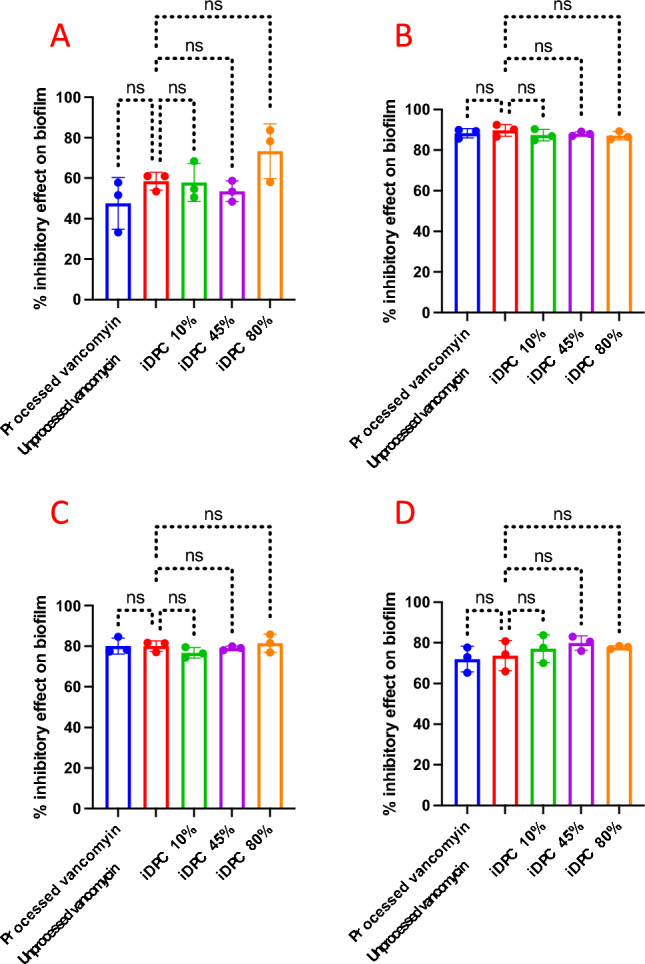
(**A**) Results of the percent of inhibitory effect on biofilm inhibition using the MIC value of 3.125 µg/mL. (**B**) Results of the percent of inhibitory effect on biofilm inhibition using the MLC value of 6.25 µg/mL. (**C**) Results of the percent of inhibitory effect on biofilm dispersal using the MIC value of 3.125 µg/mL. (**D**) Results of the percent of inhibitory effect on biofilm dispersal using the MLC value of 6.25 µg/mL. N = 3, ns = *P* > 0.05.

To evaluate the potential impact of iDPC and varying L-glutamic acid concentrations on vancomycin biofilm dispersal and inhibition, five samples were tested with two vancomycin concentrations each. These samples included iDPC-processed vancomycin, iDPC 10%, iDPC 45%, and iDPC 80% and were compared with unprocessed vancomycin (control). Both the MIC (3.125 µg/mL) and the MLC (6.125 µg/mL) concentrations of vancomycin were evaluated across the formulations, as reported in Table 2. The results depicting the percentages of dispersal and inhibition on biofilms for both MIC and MLC can be observed in Fig. 10. The results of this study indicated no significant difference in biofilm dispersal and inhibition between processed vancomycin, iDPC 10%, iDPC 45%, and iDPC 80% compared to the control, as shown in Fig. 11.

These findings are consistent with the observations made by Kolodkin-Gal et al. (2010), who reported that L-amino acids had no discernible effect on biofilm dispersal and inhibition, whereas D-amino acids can effectively inhibited and dispersed biofilms^51^. The study reports that D-amino acids promoted the disassembly of cell-surface proteins within the extracellular matrix that are responsible for the initial stage of biofilm formation, cell attachment. Conversely, biofilms in the presence of L-amino acids had no reduction in cell-surface proteins and therefore did not prevent the initial biofilm formation stage of cell attachment^51^.

The results from the MIC, MLC and biofilm dispersal and inhibition assays indicate that utilisation of the iDPC and the inclusion of L-glutamic acid does not impact the antibacterial activity of vancomycin.

## Conclusion

In conclusion, this study underscores the promising potential of buccal drug delivery for macromolecules. Initially we investigated the utility of amino acids, specifically L-glutamic acid, as adjuvants to enhance the permeation of vancomycin across the buccal cell mucosa. The introduction of L-glutamic acid yielded a significant augmentation in the permeation of vancomycin through TR146 buccal cells.

Moreover, a novel isothermal dry particle coating (iDPC) technique was employed to effectively coat vancomycin with L-glutamic acid. Investigations revealed amino acid concentration together with dry coating have a significant impact on drug permeation. Delineating the data between concentration (amino acid) and process has shown that dry particle coating process, where fine particles of glutamic acid are layered on the surface of the drug particles, results in composite particles which increase drug permeation through TR146 buccal cell layers.

Additionally, stability testing over a four-month period revealed no degradation of the drug, affirming the robustness of the formulation. Furthermore, antibacterial activity studies, including Minimum Inhibitory Concentration (MIC) and Minimum Lethal Concentration (MLC) assessments, and antibiofilm assays demonstrated that the iDPC formulation process did not compromise the antibacterial activity of vancomycin. This study not only unveils a promising avenue for macromolecule delivery through the buccal route but also offers valuable insights into optimizing the iDPC formulation process for enhanced drug permeation, ensuring drug stability, and preserving antibacterial activity.

## Materials and methods

The TR146 cell line and *Staphylococcus aureus* NCTC 10788 were obtained from Public Health England.

Vancomycin was purchased from Stratech, USA. Trifluoroacetic acid HPLC grade, L-histidine, L-glutamic acid, tryptic soy agar, tryptic soy broth, crystal violet, and phosphate buffered saline tablets were purchased from Sigma-Aldrich (Merck), UK. Hank’s balanced salt solution (HBSS), foetal bovine serum (FBS), Hams F-12 nutrient mix and trypsin–EDTA were obtained from Gibco® Lab., UK. Gentamicin and penicillin/ streptomycin were obtained from Bio Sera, UK. Acetonitrile HPLC grade, absolute ethanol, Corning Costar 12mm diameter insert, 12 well 0.4µm polycarbonate membrane tissue culture treated polystyrene plates, and Corning Costar 24-well clear TC-treated well plates were purchased from Fisher scientific, UK. All water used was Ultrapure (Type 1) Direct-Q 3 UV and autoclaved.

### High performance liquid chromatography (HPLC) assay

HPLC method development of vancomycin was adapted from Hu et al. (2022)^14^. The Agilent 1220 Infinity II LC system with UV/fluorescent detector was used, employing an Eclipse Plus column (C18 3.5µm 4.6 × 150mm). An isocratic elution using a mobile phase consisting of acetonitrile and 0.1% trifluoroacetic acid (TFA), 13:87 [v/v] was employed. The UV detection was set to 281 nm, the flow rate was 0.6 mL/min, the injection volume was set at 5 µL. Using this protocol, vancomycin eluted at approximately 8 min. This method was validated using ICH guidelines.

### Differential scanning calorimetry (DSC)

DSC was undertaken on a TA Instrument, DSCQ200 apparatus (Delaware, USA), using nitrogen as purge gas to analyse thermal properties of powders. DSC heat flow was calibrated using indium (melting point 158.47 °C). Samples weighing 3 ± 0.1 mg were sealed using Tzero pans. An empty, sealed Tzero pan was used as a reference, and each sample was ramp heated at 25 °C/minute from 25 to 250 °C to detect thermal differences. Analysis of the thermograms was performed using TA Instruments Universal Analysis 2000 version 4.5A software built in the equipment (https://www.tainstruments.com/support/software-downloads-support/instruments-by-software/). Each powder was analysed in triplicate.

### Particle size analysis

Laser diffraction was employed to measure particle size using a Sympatec HELOS/BR particle size analyser equipped with a RODOS dry dispersing system with VIBRI/I feeder (Clausthat-Zellerfield, Germany). Approximately 0.5g of sample was placed on to the VIBRI/L feeder tray and dispersed through a RODOS disperser with 3 bars of pressure. The volume mean diameter (VMD) of each sample was detected on the HELOS/BR set at a measuring range of 0–175 µm. All powders were measured in triplicate (n = 3), and the software calculate the 10% (D10), median (D50), 90% (D90) particle sizes and the volume mean diameter (VMD).

### Ball milled powders

Milled samples were prepared using a Fritsch Pulverisette 7 planetary ball mill (Idar-Oberstein, Germany). A ball to powder ratio (BPR) of 8:1 was used for all samples with varying milling speeds and times, shown in Fig. 5. Powders were accurately weighed according to the BPR. The samples were transferred into agate vials (45 cm^3^ Volume) along with 8 agate balls (10mm diameter). The vials were then sealed with a plastic ring to prevent atmospheric contamination.

### TR146 Cell Culture Procedures

TR146 cells were grown and maintained in 75 cm^3^ T-flasks in Ham’s F-12 cell culture media with the addition of 50 mL of foetal bovine serum (FBS), 2.5mL of 1% penicillin–streptomycin, 10 mL of 2 mM glutamine, 1 mL of gentamicin (10 mg/mL), and 1 mL of amphotericin B (250 µg/mL). The cells were incubated at 37 °C, 5% CO_2_ and 95% air. The media was changed every 2–3 days and when 90% confluency was reached cells were passaged using 5 mL of Trypsin–EDTA solution and seeded unto 12 well transwell inserts at a density of 24,000 cells/cm^2^. Passage number 12–15 were used for these experiments.

### Trans-epithelial electric resistance (TEER)

The ohmic resistance of cells grown on transwell inserts was measured using an EVOM3 (Epithelial Volt/Ohm Meter) with chopstick electrodes. The electrodes were placed erect, such that the longer electrode touched the basolateral chamber, while the shorter electrode touched the apical membrane chamber. TEER, which reveals the integrity of the cellular layers, was calculated from triple reading from replicate transwells (n = 9).

### In vitro transbuccal permeation studies

Permeability studies were conducted as described by Neilsen and Rassing^52^ at 37 °C and 140 rpm in an orbital plate shaker. Formulation solutions containing 450–500 µg/0.5 mL HBSS and in the presence of amino acids with concentrations ranging from 50 to 100 µg/0.5 mL, with the pH of the solutions measured before and after the permeation study. Cells on the transwell were rinsed twice with HBSS (37 °C) by adding 0.5 mL to the apical chamber and 1.5 mL to the basolateral chamber. After 2 h (duration of experiment), cells were rinsed with 0.5 mL HBSS and equilibrated for 30 min after which TEER values were measured. All samples were analysed for vancomycin content by HPLC.

### Scanning electron microscopy (SEM)

SEM micrographs were obtained using the Environmental Scanning Electron Microscope mode of the JSM-IT200 InTouchScope™ microscope equipped with a field emission filament (FEG). Approximately 1mg of each sample was adhered to a double-sided adhesive strip and placed on to an aluminium stub, and were sputter coated with platinum. Images were taken in high vacuum mode, with acceleration of 5 and kV and 3 spot size. The images allow exploration of particle size analysis, particle morphology and potential coating of L-glutamic acid on vancomycin.

### Determination of the minimum inhibitory concentration (MIC) and minimum lethal concentration (MLC) of vancomycin and vancomycin -L-glutamic acid formulations

MIC and MLC studies of vancomycin and vancomycin-L-glutamic acid formulations were undertaken on Staphylococcus aureus NCTC 10,788. Overnight cultures of S. aureus were grown by inoculating a bacterial colony in 10 mL sterile TSB, incubated overnight at 37 °C in a shaking incubator. The overnight culture (1 × 109 bacterial cells/mL) was 100 folds diluted to achieve a concentration of 1 × 107 bacterial cells/mL.

10 sterile tubes were labelled from 1 to 10, followed by aseptic transfer of 5 mL of sterile TSB into each tube. Vancomycin and each vancomycin-L-glutamic acid formulations were prepared at 25 µg/mL vancomycin concentration. 5 mL of a particular sample (Vancomycin and vancomycin-L-glutamic acid formulations) was aseptically added to 5 mL TSB in tube 1, and vortexed. 5 mL solution from tube 1 was then aseptically added to 5 mL TSB in tube 2, and thoroughly mixed and vortexed. This process was repeated for tubes 3–8. Tubes 9 and 10 were used as positive and negative controls, respectively. Once the process of dilution was completed, 100 µL from the 100 folds diluted S. aureus overnight culture was aseptically transferred into tunes 1–9, and incubated at 37 °C in a static incubator for 24 h. Tube 10 being negative control, did not have any inoculum. MICs were taken as the lowest concentrations with no turbidity (no visible bacterial growth).

Following this, the contents from the tubes with no turbidity were inoculated on Tryptic Soy Agar (TSA) plates, and incubated overnight at 37 °C in a static incubator. MLCs were taken as the lowest concentrations which resulted in no colony formation on Agar plates.

### Biofilm growth and staining

The optical density (OD600) of Staphylococcus aureus overnight culture was measured at 600 nm, and adjusted to 0.4 using a spectrophotometer. 0.8 mL of the adjusted inoculum was transferred into respective wells. The plates were incubated for 72 h at 37 °C in a static incubator. The media was removed by gentle shaking and dabbing the plates on paper towels. The wells were then washed 2–3 times with 500 µL PBS at a pH of 7.4, followed by staining with 500 µL of 0.1% crystal violet for 25–30 min. The wells were then washed by submerging the plates into a tub of distilled water to remove excess crystal violet. The plates were then allowed to dry for 3–4 h, followed by the addition of 700 µL of 30% (w/v) acetic acid to dissolve the content in each well. The plates were incubated for 20 min at room temperature, and the contents were transferred to a new 24-well plate. The OD of the biofilm was quantified using a plate reader at a 600 nm wavelength.

### Biofilm dispersal assay

Biofilms were grown for 72 h before the addition of vancomycin and vancomycin-L-glutamic acid formulations. After 72 h of incubation, the wells were washed 2–3 times with PBS at a pH of 7.4. Sample formulations at MICs and MLCs were prepared in sterile TSB. 800 µL of vancomycin and vancomycin-L-glutamic acid at MICs and MLCs were added to the respective wells. The plates were then incubated overnight at 37 °C in a static incubator, washed and stained with crystal violet, and the biofilm was quantified using a plate reader.

### Biofilm inhibition assay

The inhibitory effects of vancomycin and vancomycin-L-glutamic acid formulations on biofilms were determined by allowing the biofilms to grow whilst being exposed to vancomycin and vancomycin-L-glutamic acid formulations at MICs and MLCs. After adjusting the OD of Staphylococcus aureus overnight culture to 0.4, the formulations were added to the respective wells. The plates were then incubated for 72 h in a static incubator at 37 °C, followed by biofilm staining and quantification.

### Statistical analysis

The data was generated in triplicates, where n = 3, and analysed for statistical significance using one-way analysis of variance (ANOVA) and Dunnett's multiple comparisons post-test from GraphPad Prism® version 9.4.0. The level of significance was set to *P* < 0.05 (probability values of 95%).

### Supplementary Information

Supplementary Tables.

## Data Availability

All data generated or analysed during this study are included in this published article.

## References

[1] Anselmo, A. C., Gokarn, Y. & Mitragotri, S. Non-invasive delivery strategies for biologics. *Nat. Rev. Drug Discov.* **18**, 19–40. 10.1038/nrd.2018.183 (2019). 30498202 10.1038/nrd.2018.183

[2] Montenegro-Nicolini, M. & Morales, J. O. Overview and future potential of buccal mucoadhesive films as drug delivery systems for biologics. *AAPS PharmSciTech* **18**, 3–14. 10.1208/s12249-016-0525-z (2017). 27084567 10.1208/s12249-016-0525-z

[3] Gilhotra, R. M., Ikram, M., Srivastava, S. & Gilhotra, N. A clinical perspective on mucoadhesive buccal drug delivery systems. *J. Biomed. Res.* **28**, 81–97. 10.7555/jbr.27.20120136 (2014). 24683406 10.7555/jbr.27.20120136 PMC3968279

[4] Sattar, M., Sayed, O. M. & Lane, M. E. Oral transmucosal drug delivery – Current status and future prospects. *Int. J. Pharm.* **471**, 498–506. 10.1016/j.ijpharm.2014.05.043 (2014). 24879936 10.1016/j.ijpharm.2014.05.043

[5] Harris, D. & Robinson, J. R. Drug delivery via the mucous membranes of the oral cavity. *J. Pharm. Sci.* **81**, 1–10. 10.1002/jps.2600810102 (1992). 1619560 10.1002/jps.2600810102

[6] Iyire, A., Alaayedi, M. & Mohammed, A. R. Pre-formulation and systematic evaluation of amino acid assisted permeability of insulin across in vitro buccal cell layers. *Sci. Rep.* **6**, 32498. 10.1038/srep32498 (2016). 27581177 10.1038/srep32498 PMC5007592

[7] Veuillez, F., Kalia, Y. N., Jacques, Y., Deshusses, J. & Buri, P. Factors and strategies for improving buccal absorption of peptides. *Eur. J. Pharm. Biopharm.* **51**, 93–109. 10.1016/S0939-6411(00)00144-2 (2001). 11226816 10.1016/S0939-6411(00)00144-2

[8] Gamboa, A. *et al.* Delivery of ionizable hydrophilic drugs based on pharmaceutical formulation of ion pairs and ionic liquids. *Eur. J. Pharm. Biopharm.* **156**, 203–218. 10.1016/j.ejpb.2020.09.007 (2020). 32976927 10.1016/j.ejpb.2020.09.007

[9] Samineni, R., Chimakurthy, J. & Konidala, S. Emerging role of biopharmaceutical classification and biopharmaceutical drug disposition system in dosage form development: A systematic review. *Turk. J. Pharm. Sci.* **19**, 706–713. 10.4274/tjps.galenos.2021.73554 (2022). 36544401 10.4274/tjps.galenos.2021.73554 PMC9780568

[10] Phan, T. N. Q., Shahzadi, I. & Bernkop-Schnürch, A. Hydrophobic ion-pairs and lipid-based nanocarrier systems: The perfect match for delivery of BCS class 3 drugs. *J. Controll. Release* **304**, 146–155. 10.1016/j.jconrel.2019.05.011 (2019). 10.1016/j.jconrel.2019.05.011 31075345

[11] Tran, P. H. L., Duan, W. & Tran, T. T. D. Recent developments of nanoparticle-delivered dosage forms for buccal delivery. *Int. J. Pharm.* **571**, 118697. 10.1016/j.ijpharm.2019.118697 (2019). 31526839 10.1016/j.ijpharm.2019.118697

[12] Samiei, N. *et al.* Ion-pair strategy for enabling amifostine oral absorption: Rat in situ and in vivo experiments. *Eur. J. Pharm. Sci.* **49**, 499–504. 10.1016/j.ejps.2013.04.025 (2013). 23643735 10.1016/j.ejps.2013.04.025

[13] Sullivan, B. P., El-Gendy, N., Kuehl, C. & Berkland, C. Pulmonary delivery of vancomycin dry powder aerosol to intubated rabbits. *Mol. Pharm.* **12**, 2665–2674. 10.1021/acs.molpharmaceut.5b00062 (2015). 25915095 10.1021/acs.molpharmaceut.5b00062 PMC4943339

[14] Hu, C., Beyda, N. D. & Garey, K. W. A vancomycin HPLC assay for use in gut microbiome research. *Microbiol. Spectr.* **10**, e01688-e1621 (2022). 35536037 10.1128/spectrum.01688-21 PMC9241942

[15] Dahmash, E. Z. & Mohammed, A. R. Characterisation and surface-profiling techniques for composite particles produced by dry powder coating in pharmaceutical drug delivery. *Drug Discov. Today* **21**, 550–561. 10.1016/j.drudis.2015.11.013 (2016). 26689131 10.1016/j.drudis.2015.11.013

[16] Koner, J., Ershad, A., & Wyatt, D. Isothermal dry particle coating—back to the future? *ONdrugdelivery*, 28–32 (2023).

[17] Brown, T. L. *et al. Chemistry: The Central Science*. (Pearson Higher Education AU, 2013).

[18] Iyire, A., Alaayedi, M. & Mohammed, A. R. Pre-formulation and systematic evaluation of amino acid assisted permeability of insulin across in vitro buccal cell layers. *Sci Rep* **6**, 32498. 10.1038/srep32498 (2016). 27581177 10.1038/srep32498 PMC5007592

[19] Vadgama, J. V. & Evered, D. F. Absorption of amino acids from the human mouth. *Amino Acids* **3**, 271–286. 10.1007/bf00806002 (1992). 24193127 10.1007/bf00806002

[20] Rajagopal, K. A. I. & Tan, T. Book: Biochemistry free for all (Ahern, Rajagopal, and Tan).

[21] Hunt, I. S., R. 28 Chapters (Department of Chemistry, University of Calgary, 2006).

[22] Pandit, N. K. *Introduction to the Pharmaceutical Sciences: An Integrated Approach* 2nd edn. (Lippincott Williams and Wilkins, Philadelphia, 2011).

[23] Forrester, J. V., Dick, A. D., McMenamin, P. G., Roberts, F. & Pearlman, E. General and ocular pharmacology. In *The Eye* (ed. Forrester, J. V.) 338-369.e1 (Elsevier, Philadelphia, 2016).

[24] Lyrie, A. *Buccal transmucosal delivery of large molecule therapeutics using orally disintegrating tablet technology* PhD Thesis thesis, Aston University, (2016).

[25] Cristofoli, M., Kung, C. P., Hadgraft, J., Lane, M. E. & Sil, B. C. Ion pairs for transdermal and dermal drug delivery: A review. *Pharmaceutics*10.3390/pharmaceutics13060909 (2021). 34202939 10.3390/pharmaceutics13060909 PMC8234378

[26] Aulton, M. E. & Taylor, K. *Aulton’s pharmaceutics: The design and manufacture of medicines* (Elsevier, Churchill Livingstone, 2013).

[27] Guzzo, P. L., Marinho de Barros, F. B., Soares, B. R. & Santos, J. B. Evaluation of particle size reduction and agglomeration in dry grinding of natural quartz in a planetary ball mill. *Powder Technol.* **368**, 149–159. 10.1016/j.powtec.2020.04.052 (2020). 10.1016/j.powtec.2020.04.052

[28] Vollath, D. Criteria ruling particle agglomeration. *Beilstein J Nanotechnol* **12**, 1093–1100. 10.3762/bjnano.12.81 (2021). 34650901 10.3762/bjnano.12.81 PMC8491710

[29] Wang, H. & Shadman, F. Effect of particle size on the adsorption and desorption properties of oxide nanoparticles. *AIChE J.*10.1002/aic.13936 (2013). 10.1002/aic.13936

[30] Wang, H. *et al.* Formulation and particle size reduction improve bioavailability of poorly water-soluble compounds with antimalarial activity. *Malar. Res. Treat.* **2013**, 769234. 10.1155/2013/769234 (2013). 23766925 10.1155/2013/769234 PMC3666196

[31] Tipper, M. & Guillemois, E. Developments in the use of nanofibres in nonwovens. In *Advances in Technical Nonwovens* (ed. Kellie, G.) 115–132 (Woodhead Publishing, Cambridge, 2016).

[32] Aw, T. G., Gin, K. Y. H., Goh, S. G. & Te, S. H. Sample preparation of microbial contaminants in water. In *Comprehensive Sampling and Sample Preparation* (ed. Pawliszyn, J.) 723–742 (Academic Press, Cambridge, 2012).

[33] Ma, L., Wei, L., Pei, X., Zhu, X. & Xu, D. CFD-DEM simulations of particle separation characteristic in centrifugal compounding force field. *Powder Technol.* **343**, 11–18. 10.1016/j.powtec.2018.11.016 (2019). 10.1016/j.powtec.2018.11.016

[34] Miyazaki, Y., Uchino, T. & Kagawa, Y. Compounding of low-dose pharmaceutical powders using a planetary centrifugal mixer. *J. Drug Deliv. Sci. Technol.* **52**, 103–109. 10.1016/j.jddst.2019.04.007 (2019). 10.1016/j.jddst.2019.04.007

[35] Mittal, B. in *How to Develop Robust Solid Oral Dosage Forms from Conception to Post-Approval* (ed Bhavishya Mittal) 69–95 (Academic Press, 2017).

[36] Mittal, B. in *How to Develop Robust Solid Oral Dosage Forms from Conception to Post-Approval* (ed Bhavishya Mittal) 97–123 (Academic Press, 2017).

[37] Huynh-Ba, K. & Zahn, M. in *Handbook of Stability Testing in Pharmaceutical Development: Regulations, Methodologies, and Best Practices* (ed Kim Huynh-Ba) 21-41 (Springer New York, 2009).

[38] Holmes, N. E., Johnson, P. D. R. & Howden, B. P. Relationship between vancomycin-resistant *Staphylococcus aureus*, vancomycin-intermediate *S. aureus*, high vancomycin MIC, and outcome in serious *S. aureus* infections. *J. Clin. Microbiol.* **50**, 2548–2552. 10.1128/jcm.00775-12 (2012). 22593595 10.1128/jcm.00775-12 PMC3421495

[39] Kowalska-Krochmal, B. & Dudek-Wicher, R. The minimum inhibitory concentration of antibiotics: Methods, interpretation, clinical relevance. *Pathogens*10.3390/pathogens10020165 (2021). 33557078 10.3390/pathogens10020165 PMC7913839

[40] Barry, A. L. & Lasner, R. A. In-vitro methods for determining minimal lethal concentrations of antimicrobial agents. *Am. J. Clin. Pathol.* **71**, 88–92. 10.1093/ajcp/71.1.88 (1979). 105628 10.1093/ajcp/71.1.88

[41] Clinical & Institute, L. S. 106–112 (Clinical and Laboratory Standards Institute Wayne, PA, 2017).

[42] Evans, R. C. & Holmes, C. J. Effect of vancomycin hydrochloride on *Staphylococcus epidermidis* biofilm associated with silicone elastomer. *Antimicrob. Agents Chemother.* **31**, 889–894. 10.1128/aac.31.6.889 (1987). 3619420 10.1128/aac.31.6.889 PMC284205

[43] Flemming, H.-C. & Wingender, J. The biofilm matrix. *Nat. Rev. Microbiol.* **8**, 623–633. 10.1038/nrmicro2415 (2010). 20676145 10.1038/nrmicro2415

[44] Warraich, A. A. *et al.* Evaluation of anti-biofilm activity of acidic amino acids and synergy with ciprofloxacin on Staphylococcus aureus biofilms. *Sci. Rep.* **10**, 9021. 10.1038/s41598-020-66082-x (2020). 32488138 10.1038/s41598-020-66082-x PMC7265346

[45] Muhammad, M. H. *et al.* Beyond risk: Bacterial biofilms and their regulating approaches. *Front. Microbiol.*10.3389/fmicb.2020.00928 (2020). 32508772 10.3389/fmicb.2020.00928 PMC7253578

[46] Kostakioti, M., Hadjifrangiskou, M. & Hultgren, S. J. Bacterial biofilms: Development, dispersal, and therapeutic strategies in the dawn of the postantibiotic era. *Cold Spring Harb. Perspect. Med.* **3**, a010306. 10.1101/cshperspect.a010306 (2013). 23545571 10.1101/cshperspect.a010306 PMC3683961

[47] Mukherjee, S. & Bassler, B. L. Bacterial quorum sensing in complex and dynamically changing environments. *Nat. Rev. Microbiol.* **17**, 371–382. 10.1038/s41579-019-0186-5 (2019). 30944413 10.1038/s41579-019-0186-5 PMC6615036

[48] Solano, C., Echeverz, M. & Lasa, I. Biofilm dispersion and quorum sensing. *Curr. Opin. Microbiol.* **18**, 96–104. 10.1016/j.mib.2014.02.008 (2014). 24657330 10.1016/j.mib.2014.02.008

[49] Mendhe, S., Badge, A., Ugemuge, S. & Chandi, D. Impact of biofilms on chronic infections and medical challenges. *Cureus* **15**, e48204. 10.7759/cureus.48204 (2023). 38050493 10.7759/cureus.48204 PMC10693677

[50] Davies, D. Understanding biofilm resistance to antibacterial agents. *Nat. Revi. Drug Discov.* **2**, 114–122. 10.1038/nrd1008 (2003). 10.1038/nrd1008 12563302

[51] Kolodkin-Gal, I. *et al.* D-amino acids trigger biofilm disassembly. *Science* **328**, 627–629. 10.1126/science.1188628 (2010). 20431016 10.1126/science.1188628 PMC2921573

[52] Nielsen, H. M. & Rassing, M. R. TR146 cells grown on filters as a model of human buccal epithelium: III. Permeability enhancement by different pH values, different osmolality values, and bile salts. *Int. J. Pharm.* **185**, 215–225. 10.1016/s0378-5173(99)00165-9 (1999). 10460917 10.1016/s0378-5173(99)00165-9

