# Supplementary material for: Evaluating the role of amino acids and isothermal dry particle coating in modulating buccal permeation of large molecule drug vancomycin
Source: Sci Rep. 2024 Aug 24;14:19678. doi: 10.1038/s41598-024-69144-6 (PMC11344821; doi:10.1038/s41598-024-69144-6)
Supplement: Supplementary file 1 — Supplementary Tables. [file 41598_2024_69144_MOESM1_ESM.docx]

Evaluating the Role of Amino Acids and Isothermal Dry Particle Coating in Modulating Buccal Permeation of Large Molecule Drug Vancomycin

Anthony Rajabi^1^, Muhammed Idrees^1^, Ayesha Rahman^2,4^, Affiong Iyire^1^, David Wyatt^3^, Jasdip Koner^3^, Afzal R Mohammed^1*^

^1^Aston Pharmacy School, College of Health and Life Sciences, Aston University, Birmingham, UK

^2^ School of Healthcare, University of Leicester, Leicester, UK

^3^Aston Particle Technologies Ltd, Birmingham, UK

^4^Dentistry, School of Health Sciences, College of Medicine and Health, University of Birmingham, UK

*Corresponding author

Aston Pharmacy School

Aston University

Birmingham

B4 7ET

Email: a.u.r.mohammed@aston.ac.uk

Table S1 Particle size distribution analysis of vancomycin, and L-glutamic acid using laser diffraction (n=3).

Table S2 Particle size analysis of L-glutamic acid (G2 – 15-minute run time, 400 rpm, and 8 ball to powder ratio) completed on day 0, day 1, day 3, day 7, day 14, and day 28. The particle size analysis showed that there was no agglomeration, indicating stability of the ball milled L-glutamic acid over the course of 28 days.
